# Scalable reaction network modeling with automatic validation of consistency in Event-B

**DOI:** 10.1038/s41598-022-05308-6

**Published:** 2022-01-25

**Authors:** Usman Sanwal, Thai Son Hoang, Luigia Petre, Ion Petre

**Affiliations:** 1 Mälardalen University, Västerås, Sweden; 2 School of Electronics and Computer Science, University of Southampton, Southampton, UK; 3 Computer Science, Abo Akademi University, 20500 Turku, Finland; 4 Department of Mathematics and Statistics, University of Turku, 20014 Turku, Finland; 5 National Institute for Research and Development in Biological Sciences, 060031 Bucharest, Romania

**Keywords:** Biochemical reaction networks, Computational models, Networks and systems biology

## Abstract

Constructing a large biological model is a difficult, error-prone process. Small errors in writing a part of the model cascade to the system level and their sources are difficult to trace back. In this paper we extend a recent approach based on Event-B, a state-based formal method with refinement as its central ingredient, allowing us to validate for model consistency step-by-step in an automated way. We demonstrate this approach on a model of the heat shock response in eukaryotes and its scalability on a model of the $\mathsf {ErbB}$ signaling pathway. All consistency properties of the model were proved automatically with computer support.

## Introduction

**Figure 1:**
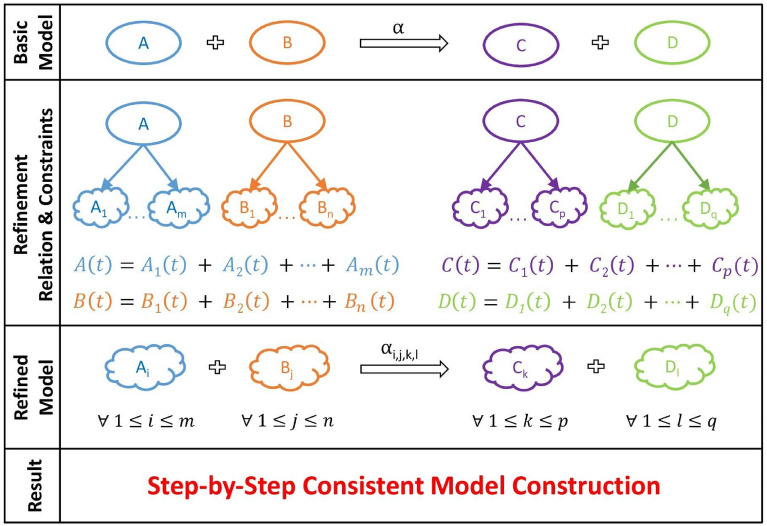
The step-by-step refinement-based approach to building a reaction network model. (Basic Model) We start with a simplified, abstract version of the model. (Refinement) We then gradually add details to the variables of the model. (Refined model) This leads to a more detailed, refined version of the reactions of the model. (Refinement constraints) The refined model is set up to ensure its consistency.

Biological processes are large, complex, concurrent systems of biochemical reactions. It is remarkably difficult to capture all the necessary details of such a system in a single all-encompassing modeling step^1^: details that are critical in some parts of the model are neutral in other parts of it; modules build upon each other in a structured way; there can be several levels of detail. An effective solution that has been proposed for this problem^2–7^ is to use model refinement: gradually adding details to a model while preserving its consistency. This splits the modeling processes into two stages: build first a simplified, abstract version of the model (and verify/ensure its consistency) and then add details to it step-by-step in a way that ensures that the model remains consistent. At each refinement step, one can focus only on the new elements that are introduced and on their consistency with the previous model (Fig. 1). This approach allows to also separate the reasoning about the system under development into smaller steps. Model refinement has been introduced in biomodeling in several different frameworks, such as ODE-based modeling^8^, Boolean networks^7^, process algebras^3,9^, rule-based modeling^5,10^, and Petri nets^11–13^. The key challenge in deploying this method in practice is verifying the consistency of the initial/basic model and ensuring that the model remains consistent in each step of the refinement. This is very difficult as the model size increases to hundreds/thousands of variables influencing each other through concurrent processes. A small error in writing a part of the model (say, in a variable name or in the multiplicity of a reactant or of a product) cascades into system-level inaccuracies between the basic and the refined versions of the model, whose sources are very difficult to identify. There are several other approaches to axiomatic network generation, based on graph theory, including the reaction mechanisms generator^14,15^ and the automated reaction generation^16,17^.

To address the problem of ensuring the model consistency throughout the construction of the model we recently proposed^18^ using Event-B for biomodeling. Event-B^19^ is a formal method for system modeling, with its original motivations rooted in the specifications of complex software systems. It is based on set theory, with refinement at its core, and with a focus on mathematical proofs that the different refinement levels of a model are consistent. Crucially, proving the consistency is done in Event-B at the level of its basic events, rather than on the system as a whole. This allows the modeler to identify easily the source of model inconsistencies in the events whose proof obligations fail to get discharged. Event-B is accompanied by the software toolset Rodin^20^.

In this paper we extend the Event-B-based modeling technique^18^ to give the first scalable demonstration of Event-B-based biomodeling. We show that simple mathematical functions can be used to hide some of the details of the refinement, leading to a reduction in the number of events of the model. We show that a model of the heat shock response can be described through 17 events, instead of the 57 events in an earlier model^18^. More drastically, we show that a model of the $\mathsf {ErbB}$ signaling pathway consisting of 1320 reactions can be written through only 242 events. The models were implemented using Rodin^20^ and all proof obligations related to demonstrating the model consistency were automatically discharged in Rodin. All Event-B models discussed in the paper are available at^21^.

## Results

### From a reaction network to an Event-B model

We model reaction networks as sets of biochemical reactions, where each reaction specifies its reactants, products, and possibly inhibitors and catalyzers. For simplicity, we consider each reversible reaction in our methodology as two irreversible reactions. With these assumptions, a reaction *r* can be written as a rewriting rule of the form:

1 $$\begin{aligned} r: \ \ \ m_1X_1 + m_2X_2 + \cdots + m_nX_n \rightarrow m'_1X_1 + m'_2X_2 + \cdots + m'_nX_n, \end{aligned}$$

where $\mathcal{S} = \{X_1,\ldots ,X_n\}$ is the set of *reactants* and $m_1, \ldots , m_n, m'_1, \ldots , m'_n \in \mathbb {N}$ are non-negative integers.

Reaction networks can be modeled straightforwardly in Event-B^18^: every reactant is modeled by a variable and every reaction is modeled by an event. Invariants ensure the consistency of the model as well as other biological properties of interest, for instance the mass conservation rule that requires that the number of certain reactants remains constant in the system. The general form of an Event-B model corresponding to reaction (1) is presented in Table 1; more reactions in the network model lead to more events in the Event-B model^18^. $X_1, X_2$ $, \ldots ,X_n$ are the variables of the model, whose initial values are set in the initialization event. For each reaction *r* of the reaction network, we specify in its guard that it must have enough of each reactant in order for the reaction to be enabled, while the action of the event specifies the changes to happen to each variable.

**Table 1:**
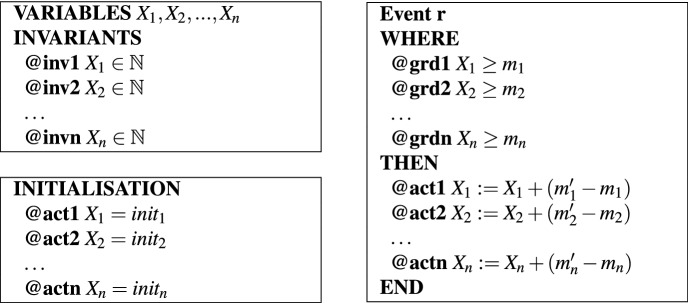
The general form of an Event-B model for a reaction network.

The event *r* models reaction (1), implementing the result of that reaction being triggered.

The data refinement of a model is about adding details to it by replacing generic variables with more specific versions of them. The refined model can include the reactions of the basic model where the refined variables replace the generic ones in all (or some) possible combinations. New reactions between the refined variables can also be added. Obviously, this easily leads to a combinatorial explosion in the size of the refined model. To see this, consider a binary binding reaction $A+B\rightarrow C$. When variable *A* is refined into two specific versions of it $\{A_0,A_1\}$, *B* into $\{B_0,B_1,B_2\}$, this leads to a refinement of *C* into $\{C_{i,j}\mid i=0,1, j=0,1,2\}$. The reaction is replaced by six refined versions of it: $A_i+B_j\rightarrow C_{i,j}$, for all $0\le i\le 1$, $0\le j\le 2$.

The case of a homogeneous binary reaction (also called a dimerization reaction) is similar: reaction $2A\rightarrow C$ and a refinement of *A* into $\{A_0,A_1\}$ leads to a refinement of *C* into $\{C_0,C_1,C_2\}$ and three refined versions of the reaction: $A_i+A_j\rightarrow C_{i+j}$, with $i,j\in \{0,1\}$.

### Size-preserving reaction network refinement in Event-B

We propose in this paper a new approach to reaction network refinement in Event-B that keeps the number of variables and events unchanged. To refine a variable *X* into *l* versions of it $\{X_1,X_2,\ldots ,X_l\}$ we replace the integer variable *X* with a function *rX* defined on a domain set partitioned into *l* singleton sets $\{X_1\}$, $\{X_2\}$, $\ldots$, $\{X_l\}$ and taking integer values. Distinguishing between the *l* versions of variable *X* can be done by checking the argument of function *rX* for the partition it belongs to. All of these are easily implementable in Event-B. The complexity of the refinement is transferred in this way to using functions defined on partitioned sets, rather than using integer variables, and in specifying more complex event guards to check the argument of the function. Crucially, this makes writing large refined models possible and it leaves checking the model consistency feasible, at least on the $\mathsf {ErbB}$ model we tested on, with more than 1000 reactions in its refined version. We discuss this technique in concrete details in the case of the binary reactions: binding $A+B\rightarrow C$ and dimerization $2A\rightarrow C$.

Consider the binding reaction $A + B \rightarrow C$. Assume that the variable *A* is refined into two versions, $A_0$ and $A_1$ and the variable *C* is refined into $C_0,C_1$. The binding reaction is then refined into two versions of it $A_i+B\rightarrow C_i$, with $i=0,1$. Rather than writing two events in the refined Event-B model, one for each of them, we can write a single encompassing event in the following way.

We replace variables *A* and *C* with the functions $rA:\{\{A_0\}, \{A_1\}\}\rightarrow \mathbb {N}$ and $rC:\{\{C_0\}, \{C_1\}\}\rightarrow \mathbb {N}$, resp., where $A_0,A_1,C_0,C_1$ are new constants. The refinement of the binding reaction can be described through a single event, shown in Table 2. The key observation is that distinguishing between the two refinements of the reaction is done in a uniform way through the guards of the event. The main guard in this case is **@grd3**, which models that the specific form $A_0$ of variable *A* corresponds to the specific form $C_0$ of variable *C* and that $A_1$ corresponds to $C_1$. These two cases are covered in **@grd3** through parameters *a* and *c*, a technique that allows a uniform specification of the two refined versions of the reaction through **@act1** and **@act3**. The gluing invariants specifying the conditions for the consistency of this refinement are $A = rA(A_0) + rA(A_1)$ and $C = rC(C_0) + rC(C_1)$.

**Table 2:**
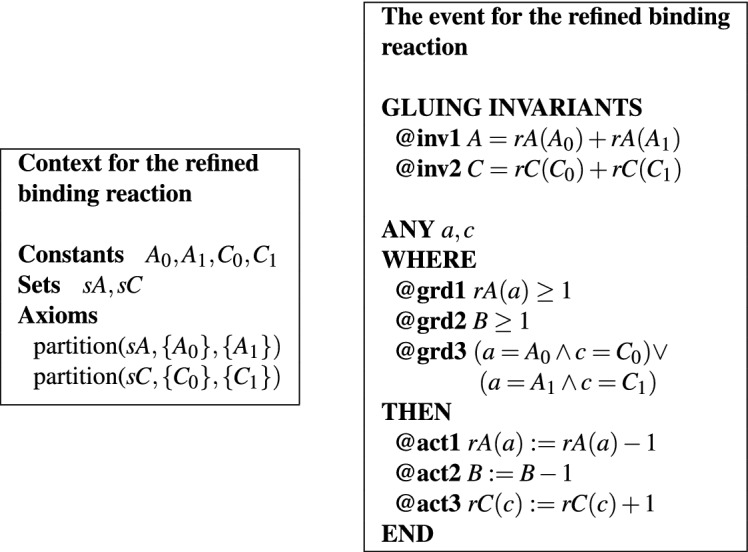
Size-preserving refinement of a binding reaction in Event-B.

A dimerization reaction $2A \rightarrow C$ can be refined in the same way.

Assume that the variable *A* is refined into two versions $A_0$ and $A_1$ and the variable *C* is refined into three versions $C_0$, $C_1$, and $C_2$. The dimerization reaction is then refined into three versions of it $A_i+A_j\rightarrow C_{i+j}$, with $0\le i\le j\le 1$. All three of them can be described through a single event, shown in Table 3.

**Table 3:**
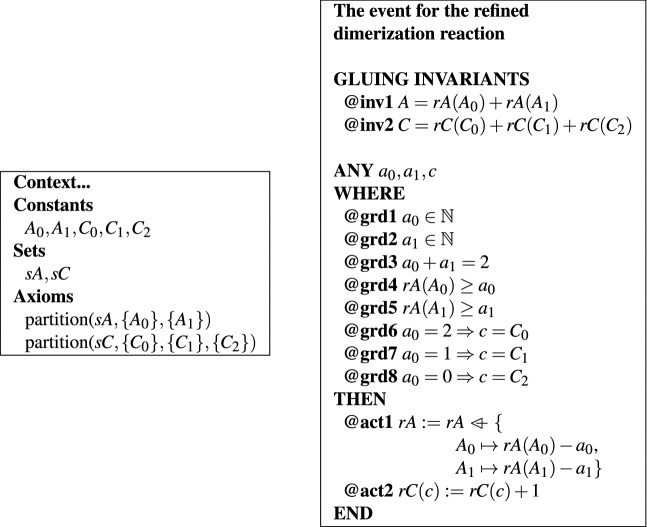
Size-preserving refinement of a dimerization reaction in Event-B.

The symbol $\mathbin {\lhd -}$ is used in Event-B to specify that the function *rA* is modified for its arguments $A_0$ and $A_1$.

The event is written similarly as for the binding reaction: variable *A* is replaced with a function $rA:\{\{A_0\}, \{A_1\}\}\rightarrow \mathbb {N}$ and variable *C* is replaced by function $rC:\{\{C_0\}, \{C_1\}, \{C_2\}\}\rightarrow \mathbb {N}$. The consistency of the refinement is specified through the gluing invariants shown in Table 3.

### A compact Event-B model for the heat shock response

The heat shock response is a well conserved cellular mechanism to react to sudden increases in temperature, in an effort to protect the cell against damage to cellular structures and essential functions^22^. It also has a key role in oncogenesis and cell death^23,24^. The reaction network model^25^ describing the heat shock response is shown in Table 4 and it consists of 10 variables and 17 irreversible reactions. It was described through an Event-B model^18^ with each (uni-directional) reaction leading to its own event in the model. We are interested in a refinement of this model where the heat shock factor ($\mathsf {hsf}$) variable is replaced with two variants $\mathsf {\mathsf {rhsf}}^{(0)}$ and $\mathsf {\mathsf {rhsf}}^{(1)}$, corresponding to the phosphorylation status of site S230 of the heat shock factor. Consequently, this leads us to also introduce simultaneously the refinement of all the other forms of $\mathsf {hsf}$:

$$\begin{aligned}&\mathsf {hsp:hsf}\rightarrow \{\mathsf {\mathsf {\mathsf {hsp}:\mathsf {rhsf}}}^{(0)}, \mathsf {\mathsf {\mathsf {hsp}:\mathsf {rhsf}}}^{(1)}\},\\&\mathsf {hsf}_{2}\rightarrow \{\mathsf {\mathsf {rhsf}}_{2}^{(0)}, \mathsf {\mathsf {rhsf}}_{2}^{(1)}, \mathsf {\mathsf {rhsf}}_{2}^{(2)}\},\\&\mathsf {hsf}_{3}\rightarrow \{\mathsf {\mathsf {rhsf}}_{3}^{(0)}, \mathsf {\mathsf {rhsf}}_{3}^{(1)}, \mathsf {\mathsf {rhsf}}_{3}^{(2)}, \mathsf {\mathsf {rhsf}}_{3}^{(3)}\},\\&\mathsf {hsf}_{3}:hse\rightarrow \{\mathsf {\mathsf {rhsf}}_{3}^{(0)}:\mathsf {hse}, \mathsf {\mathsf {rhsf}}_{3}^{(1)}:\mathsf {hse}, \mathsf {\mathsf {rhsf}}_{3}^{(2)}:\mathsf {hse}, \mathsf {\mathsf {rhsf}}_{3}^{(3)}:\mathsf {hse}\}. \end{aligned}$$

This refinement leads to 22 variables and 57 reactions in the reaction network model. We avoid this increase in the model size using our approach: the refined model can be written in Event-B through 10 variables and 17 events, the same as in the basic model. All proof obligations were discharged automatically in Rodin (some of them required a change of the default theorem prover).

**Table 4:** The molecular model for the eukaryotic heat shock response proposed in ^25^.

(1)	$2 \mathsf {hsf}\rightleftarrows \mathsf {hsf}_{2}$	(7)	$\mathsf {hsp}+\mathsf {hsf}_{3}\rightarrow \mathsf {hsp:hsf}+2 \mathsf {hsf}$
(2)	$\mathsf {hsf}+\mathsf {hsf}_{2}\rightleftarrows \mathsf {hsf}_{3}$	(8)	$\mathsf {hsp}+\mathsf {hsf}_{3}:hse\rightarrow \mathsf {hsp:hsf}+2 \mathsf {hsf}+\mathsf {hse}$
(3)	$\mathsf {hsf}_{3}+\mathsf {hse}\rightleftarrows \mathsf {hsf}_{3}:hse$	(9)	$\mathsf {hsp}\rightarrow \emptyset$
(4)	$\mathsf {hsf}_{3}:hse\rightarrow \mathsf {hsf}_{3}:hse+ \mathsf {hsp}$	(10)	$\mathsf {prot}\rightarrow \mathsf {mfp}$
(5)	$\mathsf {hsp}+\mathsf {hsf}\rightleftarrows \mathsf {hsp:hsf}$	(11)	$\mathsf {hsp}+\mathsf {mfp}\rightleftarrows \mathsf {hsp:mfp}$
(6)	$\mathsf {hsp}+\mathsf {hsf}_{2}\rightarrow \mathsf {hsp:hsf}+ \mathsf {hsf}$	(12)	$\mathsf {hsp:mfp}\rightarrow \mathsf {hsp}+\mathsf {prot}$

The comparison between the size of the basic and the refined variants of the $\mathsf {HSR}$ model is in Table 5. The full model can be downloaded at^21^.

**Table 5:** The heat shock response and the $\mathsf {ErbB}$ models statistics.

	Heat shock response models		ErbB signaling pathway models	
	Basic^25^	Refined^48^	Basic^29^	Refined^29^
Molecular species	10	20	110	394
Molecular reactions	17	57	242	1320
Variables	10	10	110	110
Events	17	17	242	242
Invariants	13	18	110	110
Proof obligations discharged by the default prover	79	73	794	15
Proof obligations discharged by other internal provers	0	62	154	1260

The number of variables and events is the same for the basic and the refined model. Some of the proof obligations of the refined model required another internal theorem prover than the default one to get them automatically discharged.

### A compact Event-B model for the ErbB signaling pathway

The $\mathsf {ErbB}$ signaling pathway is a very well studied evolutionary pathway, essential in the growth and expansion of organs and of the central nervous system^26,27^. Its main role is to induce, through the cellular membrane, a signal instigating the cell’s growth and differentiation. This pathway is often overly active in various types of cancer and has been used for a long time as a therapeutic target^28^.

The reaction network model^29–31^ of the $\mathsf {ErbB}$ signaling pathway consists of a basic model consisting of 242 reactions, extended then with the phosphorylation details of the epidermal growth factor receptor ($\mathsf {EGFR}$) and the epidermal growth factor ($\mathsf {EGF}$) to 1320 reactions. The basic model was described in Event-B^32^ and we extend it here to the full model. The epidermal growth factor receptor is refined into the four receptor members of the $\mathsf {ErbB}$ family and the epidermal growth factor is refined into two variants:

$$\begin{aligned}&\mathsf {EGFR}\rightarrow \{\mathsf {ErbB1}, \mathsf {ErbB2}, \mathsf {ErbB3}, \mathsf {ErbB4}\}; \\&\mathsf {EGF}\rightarrow \{\mathsf {EGF}, \mathsf {HRG}\}. \end{aligned}$$

This leads to a refinement of $\mathsf {EGF-EGFR}$ into the following eight variants:

$$\begin{aligned}&\mathsf {EGF-EGFR}\rightarrow \{\mathsf {EGF-ErbB1}, \mathsf {EGF-ErbB2}, \mathsf {EGF-ErbB3}, \mathsf {EGF-ErbB4},\\&\mathsf {HRG-ErbB1}, \mathsf {HRG-ErbB2},\mathsf {HRG-ErbB3}, \mathsf {HRG-ErbB4}\}. \end{aligned}$$

A similar refinement is also applied for their phosphorylated versions and for their homodimers.

They were described in our Event-B model through 242 events, as many as the basic model. All proof obligations were discharged automatically by Rodin (some of them required a change of the default theorem prover), with the comparison between the basic and the refined variants shown in Table 5. The full model can be downloaded at^21^.

## Discussion

Modeling complex biological processes is a challenging systems engineering task. A solution to addressing its complexity is to use refinement and start modeling from a conceptually simpler (more abstract) model that is consistent. We can then gradually add more details in a correctness-by-construction approach, so that the most detailed (concrete) model is mathematically proved to be consistent. In this approach, the challenge is to prove both the consistency of the basic model and that of the refinement process. The Event-B-based approach we propose in this paper addresses both of these challenges. Through the Rodin toolset, the models get a much needed automatic computer-aided verification of their consistency. The method we proposed, to implement the data refinement through the use of integer-valued functions, allowed us to preserve the size of the models in terms of the number of variables and events, with the increase in complexity reflected in the more complex guards, in the higher number of invariants, and in the switch from integer variables to integer-valued functions. This led to more proof obligations. Nevertheless, in the proof-of-concept examples we modeled, all the proof obligations were automatically discharged in Rodin, albeit some of them needed a switch from the default theorem prover to another one in the Rodin installation. Even in this case, no input from the user was needed in proving the model consistency.

Additional refinements and replacing refinement relations with more detailed ones can be done in the same way. They only involve replacing some integer variables with integer-valued functions as we discussed in this paper, or replacing the domain set of an integer-valued function with a larger domain set (reflecting the more detailed refinement). This means that the number of variables and events will remain the same (the number of proof obligations will increase). The refinement of the $\mathsf {ErbB}$ reaction network, going from 242 reactions to 1320 reactions, left unchanged the number of variables and events, but increased the number of proof obligations by about 50%. The number of variables and events of the model will certainly increase if other operations than refinement are used in the model construction, such as model composition or model union.

Specifying the refinement relation in a consistent manner is essential for our approach. This means that refining a variable *A* standing for a molecular species should lead to the corresponding refinements of all complexes that it is part of: complexes *A* : *B*, dimers $A_2$, trimers $A_3$, etc. Deducing the full consistent refinement relation based on specifying the refinement of a single variable can be done based on^6^ by specifying the relationship between the compound variables and the basic variables.

Scalable modeling remains a challenging problem. The approach we introduced here is a partial solution to the problem in the case of modeling based on data refinement. Event-B has most characteristics required for a mathematical modeling language to facilitate model reuse in systems biology^1^: it is modular, human-readable, it is hybrid (it allows for continuous and for discrete mathematics semantics, aspects beyond the focus of this paper), open, and declarative (but not graphical). Significant is also the bridge between the reaction network modeling and Event-B-based modeling, which brings to biomodeling a new framework for computer-aided verification of model consistency. This is relevant to a wide variety of models, beyond the two proof-of-concept examples in this paper.

## Methods and models

### Event-B

Event-B^33^ is a state-based formal method, building on the earlier formalisms the B-method^34^ and the action systems^35^. The system state in Event-B is described by the values of *variables* and the state changes are modeled using *events*. The variable types and the model properties that must hold during system execution are defined as *invariants*. The initial system state is described with a specific event named *Initialisation*. An event can contain *parameters*, a *guard* and an *action*. The guard is a logical predicate on the variables and parameters, describing the conditions under which the action is enabled to take place. The action describes the updates it yields to the variables when it is executed. If two or more events are enabled at the same time, then one is non-deterministically chosen and executed. If two events do not update each other’s variables, then they can be executed in any order and we can consider that they are executed in parallel. The variables and events in an Event-B model are contained in a *machine*, also referred to as the “dynamic part” of the model. An Event-B machine can *see* one or more *contexts*, also known as the “static part” of the model. A context contains definitions of constants, sets, as well as axioms about them. A general structure of an Event-B model, made out of machine *M* and context *C* is presented in Table 6.

**Table 6:**
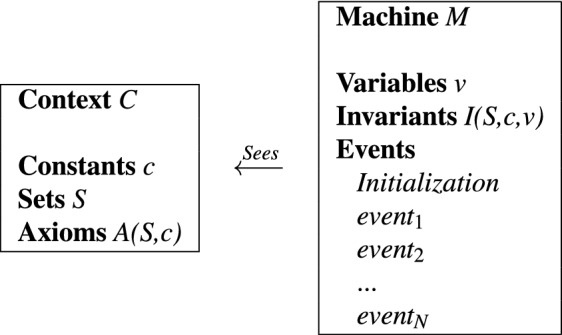
General structure of machine *M* and context *C* in Event-B.

A key concept in formal modeling with Event-B is that of *refinement*^19^, which allows the modeler to start from a simple model of the system and then gradually introduce more details, in the form of new events, variables or context data. Event-B has two types of refinements: *superposition refinement* and *data refinement*. Superposition refinement^3,4^ is the term used when new variables and events are added to the existing model. The validity of model is preserved by making sure that newly added variables and events do not contradict the previous events in any of the preceding models. In *data refinement*^2^, some variables in the abstract machine are replaced by other variables in the refined machine; in this case, a *gluing invariant* is added in the refined machine to define the relation between the abstract variables and the newly introduced, concrete ones. Refinement in Event-B has been used to model numerous protocols and systems, including smart cash card systems^36^, vehicle platoons^37^, topology discovery in graphs^38^, self-recovery in sensor-actor networks^39^, spacecraft systems^40^, coordination in peer-to-peer networks^41^, smart grid recoverability^42^, proactive routing in wireless networks^43^, reaction networks^18^, etc.

Event-B benefits from the tool support of the Eclipse-based Rodin platform^20^. Rodin allows to edit the model, to prove properties of the model, to animate the model and even allows model checking. Proving in Event-B employs several proof engines to automatically prove the different properties of the model. Rodin automatically generates all proof obligations in the form of sequents; these need to be discharged in order for the different properties (e.g., invariance, termination, or refinement) to hold. The internal provers attempt to discharge these proof obligations automatically. The remaining ones can be tackled using the interactive prover, with input from the modeler, for instance by adding extra assumptions or choosing different proof strategies. The model properties are proved at the level of the events, rather than on the systems level. Having a property that cannot be proved for some event can point out to errors in that event. The modeler then has a chance to edit the model to address the issue. Such interleaving between modeling and proving is an important aspect of working with Event-B and the Rodin platform and is quite similar to the compilation of programs^20^.

### The heat shock response

The heat-shock response is a central, well-conserved, cellular-level regulatory mechanism^44,45^. Proteins are folded in three-dimensional shapes and the fold determines whether it can achieve its functionality (e.g., bind to a certain site on a DNA molecule or on another protein). Protein folding is a dynamical process, continuously influenced by many factors, such as chemical modifications of the amino-acids forming the protein (e.g., phosphorylation, acetylation, sumoylation) and properties of the environment (e.g., temperature, radiation, heavy metals). Misfolded proteins quickly form large protein bundles that are detrimental to the normal physiology of a cell and eventually lead to cell death. The heat shock response is one of the stress response mechanisms of a cell, aiming to limit the accumulation of misfolded proteins and assisting misfolded proteins to regain their natural fold. The heat shock response synthesizes a group of proteins called heat shock proteins ($\mathsf {hsp}$s) that act as molecular chaperones for the misfolded proteins and support their recovery from stress. This is achieved either by repairing the damaged proteins or by degrading them, thus restoring protein homeostasis and promoting cell survival. Without such a mechanism, misfolded proteins will form plaque, which is the hallmark of many neurological diseases.

The basic model we discuss for the eukaryotic heat shock response^25^ is summarized in Table 4. When the temperature increases, proteins $\mathsf {prot}$ begin to misfold, namely transform into $\mathsf {mfp}$ [reaction (10)]. The heat shock proteins have a high affinity to bind to the misfolded proteins, acting as chaperones and forming $\mathsf {hsp:mfp}$ complexes [reaction (11)]. Then, the complex $\mathsf {hsp:mfp}$ can transform back into the original protein $\mathsf {prot}$, freeing the heat shock factor protein $\mathsf {hsp}$ too [reaction (12)]. The $\mathsf {hsp}$ is synthesized as follows. The heat shock factor ($\mathsf {hsf}$) binds in a trimmer form to the $\mathsf {hsp}$’s gene promoter (called the heat shock element $\mathsf {hse}$) [reactions (1)–(3) in Table 4]. The formed $\mathsf {hsf}_{3}:hse$ then produces the $\mathsf {hsp}$ proteins [reaction (4)]. These tend to combine with $\mathsf {hsf}$ and stay in inactive state as $\mathsf {hsp:hsf}$ complexes [right arrow in reaction (5), as well as reactions (6)–(8)]. Once the temperature increases and more $\mathsf {hsp}$ are becoming chaperons for $\mathsf {mfp}$, less are available for forming $\mathsf {hsp:hsf}$ complexes and the balance changes: the left arrow in the reaction (5) is activated. Finally, $\mathsf {hsp}$s can also degrade [reaction (9)].

The phosphorylation level of the heat shock factors has a key contribution to its activation and consequently to the effectiveness of the heat shock response^46^. One site in particular, S230, becomes phosphorylated only during heat shock response and drives its activity^47^. Following the model of^48^, we introduce two variants of $\mathsf {hsf}$: one where S230 is unphosphorylated and the other where S230 is phosphorylated. This cascades into several variants of all variables that $\mathsf {hsf}$ is a part of: $\mathsf {hsf}_{2}$ (phosphorylation level 0, 1, or 2), $\mathsf {hsf}_{3}$ and $\mathsf {hsf}_{3}:hse$ (phosphorylation level 0, 1, 2, or 3), and $\mathsf {hsp:hsf}$ (phosphorylation level 0 or 1). These phosphorylation-based variants replace the generic variables in all reactions, in all possible combinations, leading to an increase in the size of the model^48^.

### The ErbB signaling pathway

We discuss briefly here the key functionality of the $\mathsf {ErbB}$ signaling pathway^29–31^ using a highly simplified presentation. The epidermal growth factors ($\mathsf {EGF}$) are a family of proteins that signal to cells to grow and differentiate. They do that by binding to ligand proteins embedded in the cellular membrane—the epidermal growth factor receptors ($\mathsf {EGFR}$). Once bound, the complex dimerizes and then gets phosphorylated. This then activates other ($\mathsf {MAPK}$ and $\mathsf {ERK}$) signaling pathways. All of these activations are done step by step through a cascade of reactions, whose effect is the activation of some proteins, that then participate in other reactions activating other proteins, etc.

The model of the $\mathsf {ErbB}$ signaling pathway^29^ that we follow in this paper is a revised version of the two earlier models^30,31^. It is first presented on a more generic level, along the lines briefly described above. This initial model consists however of 242 irreversible reactions. The full model is then introduced essentially by differentiating between the four members of the $\mathsf {EGFR}$ family ($\mathsf {ErbB1}$ (also known as $\mathsf {EGFR}$), $\mathsf {ErbB2}$, $\mathsf {ErbB3}$, $\mathsf {ErbB4}$) and the two members of the $\mathsf {EGF}$ family ($\mathsf {EGF}$ and $\mathsf {HRG}$). Adding these details leads to many more variables in the model. For example, the complex $\mathsf {EGF}:\mathsf {EGFR}$ is replaced by 8 different variants of it. The full reaction network model^29^ has 1320 reactions.

## Data Availability

All models are available at^21^.
